# Effect of Thermal Properties of Aggregates on the Mechanical Properties of High Strength Concrete under Loading and High Temperature Conditions

**DOI:** 10.3390/ma14206093

**Published:** 2021-10-15

**Authors:** Taegyu Lee, Keesin Jeong, Hyeonggil Choi

**Affiliations:** 1 Department of Fire and Disaster Prevention, Semyung University, Jecheon 27136, Korea; ltg777@semyung.ac.kr (T.L.); jks@semyung.ac.kr (K.J.); 2 School of Architecture and Civil Engineering, Kyungpook National University, Daegu 41566, Korea

**Keywords:** loading and high temperature conditions, thermal properties of aggregates, high strength concrete, mechanical properties, thermal strain

## Abstract

The effect of the thermal properties of aggregates on the mechanical properties of high-strength concrete was evaluated under loading and high-temperature conditions. For the concrete, granite was selected as a natural aggregate, and ash-clay and clay as lightweight aggregates. The mechanical properties of the concrete (stress–strain, compressive strength, elastic modulus, thermal strain, and transient creep) were evaluated experimentally under uniform heating from 20 to 700 °C while maintaining the load at 0, 20, and 40% of the compressive strength at room temperature. Experimental results showed that the concrete containing lightweight aggregates had better mechanical properties, such as compressive strength and elastic modulus, than that of the concrete with a granite aggregate at high temperature. In particular, the concrete containing lightweight aggregates exhibited high compressive strength (60–80% of that at room temperature) even at 700 °C. Moreover, the concrete containing granite exhibited a higher thermal strain than that containing lightweight aggregates. The influence of the binding force under loaded conditions, however, was found to be larger for the latter type. The transient creep caused by the loading was constant regardless of the aggregate type below 500 °C but increased more rapidly when the coefficient of the thermal expansion of the aggregate was above 500 °C.

## 1. Introduction

Concrete is a non-combustible composite composed of inorganic materials, such as cement and mineral admixtures, as well as water and fine and coarse aggregates. Therefore, it has been widely used to secure the fire-resistance of structures and various experiments have been performed to predict its structural behavior more accurately in the event of fire [1,2,3,4].

To predict the behavior of concrete structures at high temperatures, the thermal properties of various materials, such as aggregates, cement paste, and admixture, must be sufficiently reflected. In general, the mechanical behavior of concrete at room temperature is explained using the elastic theory. At high temperatures, concrete exhibits a shrinking or expansion under the influence of the thermal properties of its components. It also exhibits non-linear behavior due to the influences of spalling, compressive strength and elastic modulus degradation, and short-term high-temperature creep [5,6,7,8,9,10].

Among the materials that constitute concrete, coarse aggregate accounts for 30–50% of the concrete volume, and its thermal properties significantly affect concrete. Therefore, the Comite Europeen de Normalisation (CEN) [11,12] and Comites Euro-International du Beton (CEB) codes [13] have suggested design models for fire-resistance performance of general and lightweight aggregate concrete considering the thermal properties of aggregates.

The data used in these models present criteria based on the data identified by researchers, such as Abrams [14], Castillo [5], Schneider [15], Hertz [16], and Hammer [17]. The thermal properties of concrete were mainly evaluated through unloaded residual strength test methods that did not consider loading conditions. Therefore, these data did not consider the actual environment of structures even though they are effective in evaluating behavior considering the thermal expansion properties of aggregates.

These models are mainly used for the design of fire-resistance performance because of the excessive design that considers a sufficient safety factor, and because the thermal behavior of the concrete of actual structures can differ owing to the presence of design loads. Therefore, there is an increasing demand for data of the thermal properties of concrete under loaded conditions, and researchers such as Harada, Anderberg, Sullivan, and Kodur have studied the short-term high-temperature creep (hereafter, transient creep) generated under load [18,19,20,21]. However, these studies mostly focused on the thermal properties of general strength concrete that uses carbonate and silica aggregates.

For concrete that uses lightweight aggregates, the CEB has suggested a concrete model that utilizes expanded clay under unloaded conditions. Because lightweight aggregates have different thermal properties depending on their production method or components, more data are required to understand the behavior of lightweight aggregate concrete. In addition, research on concrete behavior under loaded conditions is required because lightweight aggregates have a low strength.

Therefore, the effects of high temperatures and loading on the mechanical properties (stress–strain, compressive strength, elastic modulus, thermal strain, and transient creep) of concrete containing natural and lightweight aggregates with different thermal properties were studied here. Granite aggregate was used for the natural aggregate, and aggregates with ash-clay and clay as the main components were used for the lightweight aggregates. In addition, the effects of the thermal strain of the aggregate and the shrinkage deformation under load on the mechanical properties of concrete were analyzed.

## 2. Experimental Work

### 2.1. Materials

Table 1 and Table 2 show the physical and chemical properties of the materials used here.

Ordinary Portland cement (OPC) having a specific surface area of 3160 m^2^/g and a density of 3150 kg/m^3^ was used. SiO_2_ represented more than 90% of the silica fume (SF), which had a specific surface area of 200,000 m^2^/g and a density of 2200 kg/m^3^. For the coarse aggregates, granite was selected as the natural aggregate, and ash-clay and clay as the lightweight aggregates. The granite had a density of 2670 kg/m^3^, a maximum size of 20 mm, and an absorption of 1.0%. Furthermore, sea sand (density: 2560 kg/m^3^, FM: 2.65, absorption: 1.00%) was used as the fine aggregate. The corresponding values of the ash-clay were 1680 kg/m^3^, 13 mm, and 15.2%, while those of the clay were 1790 kg/m^3^, 13 mm, and 17.4%.

**Figure 1:**
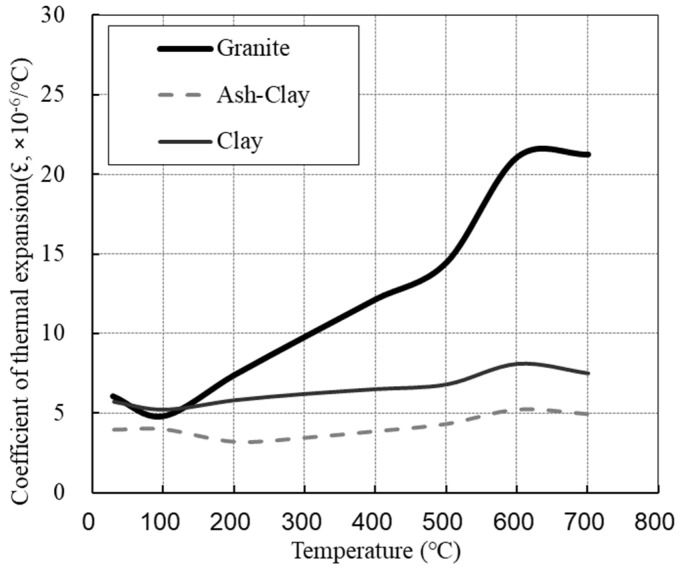
Coefficient of thermal expansion of used aggregate (1 × 10^−6^/°C).

Figure 1 shows the coefficient of thermal expansion of the coarse aggregate according to temperature, which displays ranges of 5–22 × 10^−6^/°C, 4–6 × 10^−6^/°C, and 5–8 × 10^−6^/°C for granite, ash-clay, and clay, respectively, as the temperature increased from 20 to 700 °C.

### 2.2. Experimental Plan and Mix Proportions

Table 3 presents the experimental plan for this study. A high-compressive strength concrete (60 MPa) was used. The concrete that used granite, ash-clay, and clay as the aggregate was referred to as granite concrete (GC), ash-clay concrete (AC), and clay concrete (CC), respectively. Through a preliminary test, the water-to-binder (W/B) ratios were set to 35% for the GC and 33% for the AC and CC for which similar compressive strengths were set.

The loading conditions of the concrete were set to 0.0, 0.2, and 0.4× the compressive strength. The specimens were heated at temperatures of 100–700 °C during preloading. The stress–strain, compressive strength, elastic modulus, thermal strain, and transient creep were then evaluated.

Table 4 shows the proportions of the concrete mixtures and properties of the fresh concrete. The concrete mixtures were set such that the volume of the coarse aggregate was constant. For the concrete that used the lightweight aggregates, the W/B ratio was set to a lower value, and 7% SF was added because the strength development was lower than that of the concrete that used the granite aggregate.

As for the properties of the fresh concrete, the GC exhibited a unit weight of 2410 kg/m^3^, a slump of 190 mm, and an air content of 3.3%. The corresponding values of the AC were 1958 kg/m^3^, 180 mm, and 3.5%, while those of the CC were 2031 kg/m^3^, 175 mm, and 3.6%.

### 2.3. Test Methods

#### 2.3.1. Fresh and Hardened Properties

Table 5 lists the test methods for estimating the fresh and hardened properties of the concrete. To evaluate the properties of the former, the slump was tested in accordance with ASTM C143/C143M [22], and the air content according to ASTM C231/C231M-17a [23]. Concrete specimens with dimensions of Ø100 mm × 200 mm were fabricated. The compressive strength was evaluated at planned ages in accordance with ASTM C873/C873M [24] and C39/C39M [25], and the elastic modulus was evaluated similarly in accordance with ASTM C469 [26]. The concrete specimens were cured in water for seven days and then subjected to dry curing until 180 days of age in a constant temperature and humidity chamber at 20 ± 2 °C and RH = 50 ± 5% [27].

The compressive strength of the concrete was evaluated at 28 and 180 d. Finally, the compressive strengths before the fire-resistance test were found to be 60.2 MPa for the GC, 65.3 MPa for the AC, and 52.0 MPa for the CC.

#### 2.3.2. Heating Method

**Figure 2:**
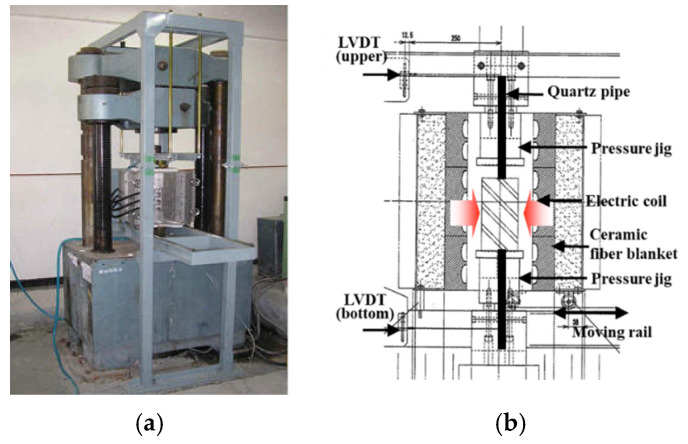
Loading system and electric furnace. (**a**) loding system. (**b**) electric furnace.

Figure 2 shows the loading system and electric furnace. A 2000 kN UTM (Shimadzu, Kyoto, Japan) was used for the loading, and an electric furnace was installed between the top and bottom pressure jigs so that the loading and heating could be performed simultaneously.

**Figure 3:**
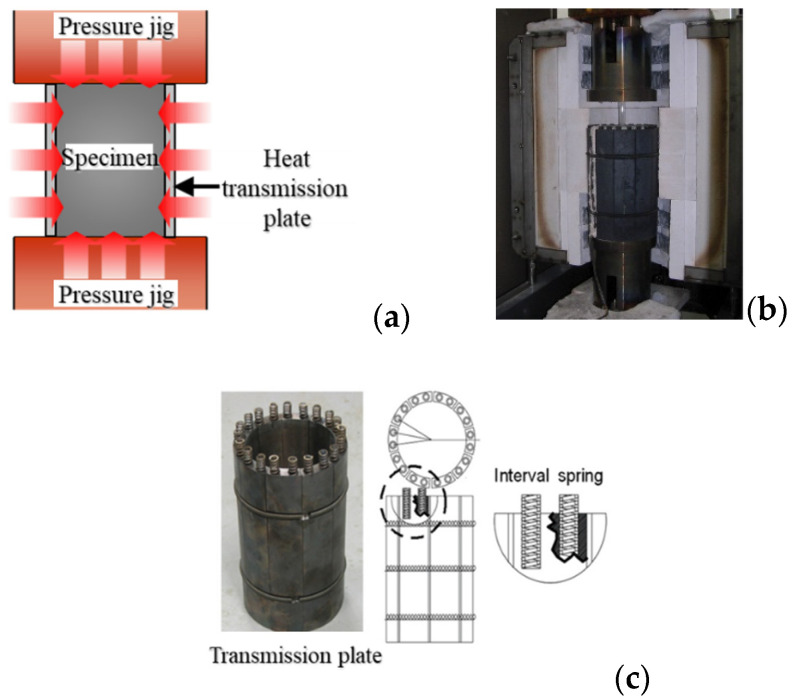
Heat transfer device; (**a**) heating flow; (**b**) transmission plate; (**c**) Transmission plate detail.

The concrete specimens were heated using a combined electric heater, as shown in Figure 3. After applying a load for three hours in advance, the pressure jigs located at the top and bottom of the furnace were heated, and the heat was transferred to the specimen. The temperature during heating was adjusted using the temperature data of the specimen obtained by using a thermocouple, temperature control function of the electric heater, and automatic programming of the relationship between the time and temperature change.

**Figure 4:**
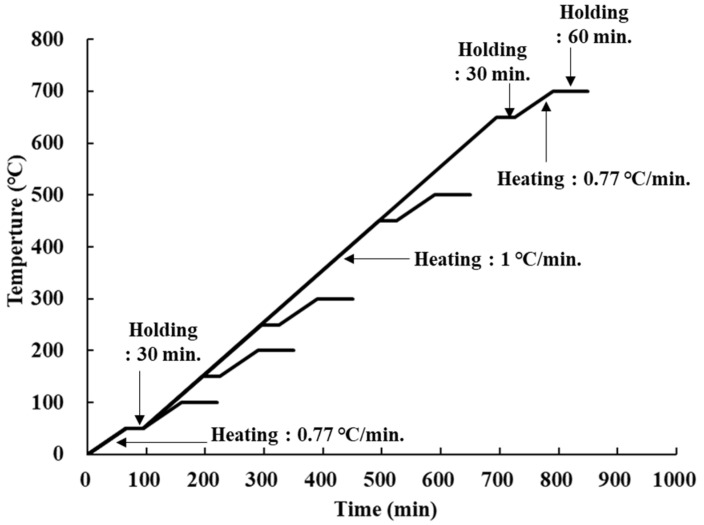
Heating curve.

As for the heating rate of the specimen, a preliminary test was conducted at a temperature below 1 °C, based on the recommendations of previous studies and RILEM TC 129-MHT [27,28,29]. Consequently, the temperature difference between the specimen inside and outside was relatively large, over 50 °C. Thus, the optimal heating curve was derived through a preliminary test considering the holding time at the target temperature, and the results are shown in Figure 4.

The concrete specimen was heated at a rate of 1 °C/min, but it was heated at a rate of 0.77 °C/min for the initial 50 °C section before reaching the target temperature, and the temperature was maintained for 30 min for heating the inside and outside uniformly. At ±50 °C of the target temperature, the heating rate was slowed to 0.77 °C/min, and the temperature was maintained for 60 min when the final temperature was reached. As a result, the temperature difference of the concrete specimen became equal to ±5 °C from the target temperature.

#### 2.3.3. Strain and Mechanical Properties under Loading and High Temperature

For the concrete specimens, the loads of 0.0, 0.2, and 0.4 fcu compared to the strength at room temperature were maintained for three hours before heating to ensure a stable application of the loads. In addition, the same loading conditions were maintained through an automatic program during heating.

The specimen displacement due to heating under load was measured using the LVDT strain gauges (Tokyo Sokki Kenkyujo Co., Tokyo, Japan) installed in the upper and lower parts after installing quartz pipes by drilling ∅15 mm holes in the center of the top and bottom pressure jigs. The maximum capacity of the LVDT strain gauges was 5 mm, and a data logger was used for recording during the experiment.

**Figure 5:**
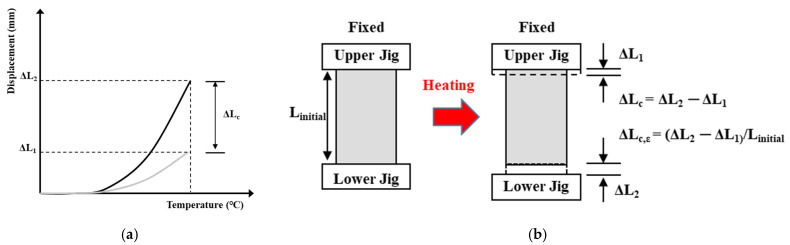
Test method for determining the thermal strain of concrete with loading and heating. (**a**) total deformation. (**b**) total strain.

Figure 5 shows the test method for determining the thermal strain of the concrete with loading and heating. The deformation and transient creep of the concrete specimen were calculated using Equations (1) and (2) in accordance with RILEM TC 129-MHT Part 6-thermal strain [28] and RILEM TC 129-MHT Part 7-transient creep [29], respectively:

(1) $$\Delta L_{C,\;\varepsilon }=(\Delta L_{2}-\Delta L_{1})/L_{initial}$$

where Δ*L*c,ε is the thermal strain of concrete; Δ*L*2 and Δ*L*1 are the displacements of the upper and lower strains gauge, respectively; and *L_iniital_* is the initial length of the specimen before heating.

(2) $${↋}_{tr}={↋}_{tot}-{↋}_{th}-{↋}_{el}$$

where *↋_tr_* is the transient creep, *↋_tot_* is the total strain (0.0, 0.2, and 0.4 fcu loaded), *↋_th_* is the thermal strain (0.0 fcu loaded), and *↋_el_* is the initial elastic deformation.

The relationships were analyzed considering the thermal properties of the aggregate and loading conditions. Equation (3) was used to analyze the relationship between the thermal strains of the aggregate and those of concrete:

(3) $${↋}_{r}=\frac{{↋}_{c,t}}{{↋}_{a,t}}$$

where *↋_r_* is the ratio of strain, *↋_c,t_* is the concrete strain at temperature *t*, and *↋_a,t_* is the thermal strain of the aggregate at temperature t.

In this study, three concrete specimens were used, and the results were calculated as an average value.

## 3. Experimental Results and Discussions

### 3.1. Stress–Strain Curve

**Figure 6:**
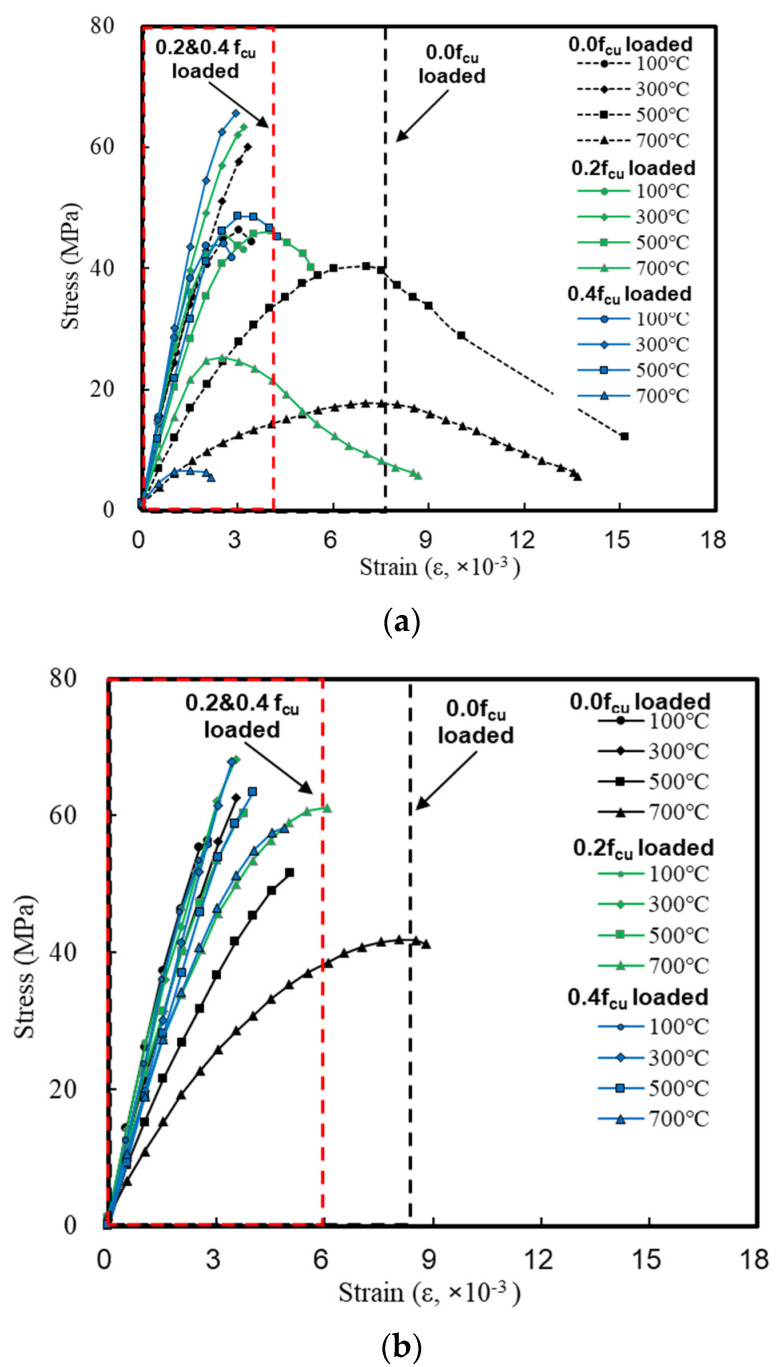

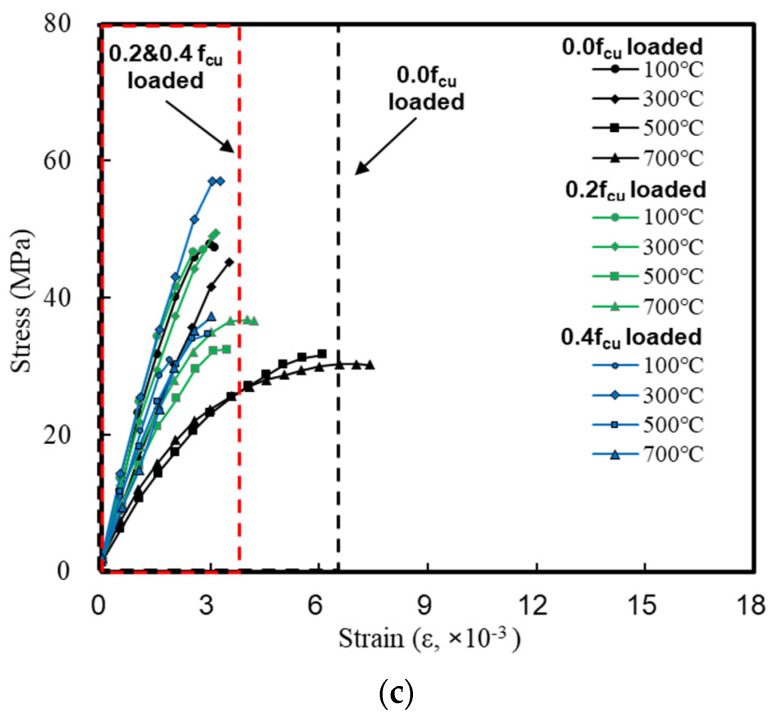
Stress–strain curve of concrete. (**a**) GC. (**b**) AC. (**c**) CC.

Figure 6 shows the stress–strain curves of the concrete according to the loading conditions. For the GC, the compressive strength was nonlinear under a load of 0.0 fcu as it decreased at 100 °C, increased at 300 °C, and decreased again after reaching 500 °C. The stress–strain curve was also nonlinear, and the strain ranged from 2.3–7.0 (ε, ×10^−3^).

When loads of 0.2 and 0.4 fcu were applied, a linear behavior was observed as the temperature increased. The stress decreased, while the slope of the stress–strain curve did not change significantly; the strain ranged from 2.3–4.5 (ε, ×10^−3^).

For the AC, the slope of the stress–strain curve decreased at 300 °C under a load of 0.0 fcu, and brittle failure was observed even at a relatively high temperature compared to the GC. When loads of 0.2 and 0.4 fcu were applied, the slope of the stress–strain curve became linear with approximately 90% of the stress remaining, and the strain ranged from 2.3–6.0 (ε, ×10^−3^).

For the CC, a brittle behavior similar to that of the AC was observed, but the residual strength tended to be somewhat lower. In addition, the strength showed a tendency to decrease in the same pattern as the GC at temperatures higher than 500 °C. Therefore, the strain of the CC was not significantly different from that of the GC even though it ranged from 2.3–7.3 (ε, ×10^−3^) under a load of 0.0 fcu and from 2.3–4.0 (ε, ×10^−3^) at 0.2 and 04 fcu.

### 3.2. Compressive Strength with Loading and Heating

**Figure 7:**
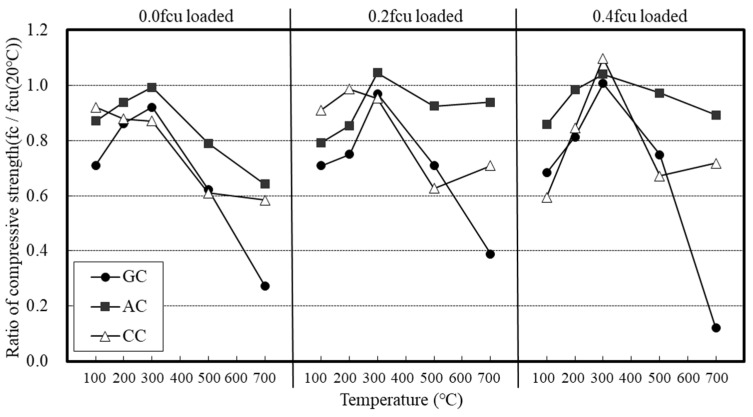
Relative compressive strength of the concrete with loading condition.

Figure 7 shows the relative compressive strength of the concrete with loading condition.

Under the loading condition of 0.0 fcu, the GC showed the lowest residual compressive strength. In the case of the concrete that used lightweight aggregates, the AC exhibited a 1.3 to 2 times higher residual compressive strength than that of the GC at 500 °C or higher.

The CC showed a larger reduction in compressive strength at 100 °C under a load of 0.0 fcu unlike the AC, and its residual compressive strength at high temperature was slightly higher even under loaded conditions. Above 300 °C, however, the residual compressive strength of the concrete was not significantly different from that of the GC.

Under the loading conditions of 0.2 and 0.4 fcu, the compressive strength of the concrete was found to decrease at all temperatures. For all the concrete specimens, the maximum compressive strength was observed at 300 °C. The concrete specimens exhibited 1–8% and 1–4% higher compressive strength than that at room temperature under the loading conditions of 0.0 fcu, and 0.2 and 0.4 fcu, respectively. Although the strength of the concrete generally decreased at temperatures higher than 300 °C, the reduction in strength tended to be smaller for the concrete specimens under load, and the AC exhibited a very small reduction in strength.

In general, the compressive strength tended to decrease at high temperatures; however, it was confirmed that the strength tended to increase slightly in the range of 200–300 °C. This can be ascribed to the stress generated by the water vapor pressure or the thermal expansion of the aggregate [16,17].

**Figure 8:**
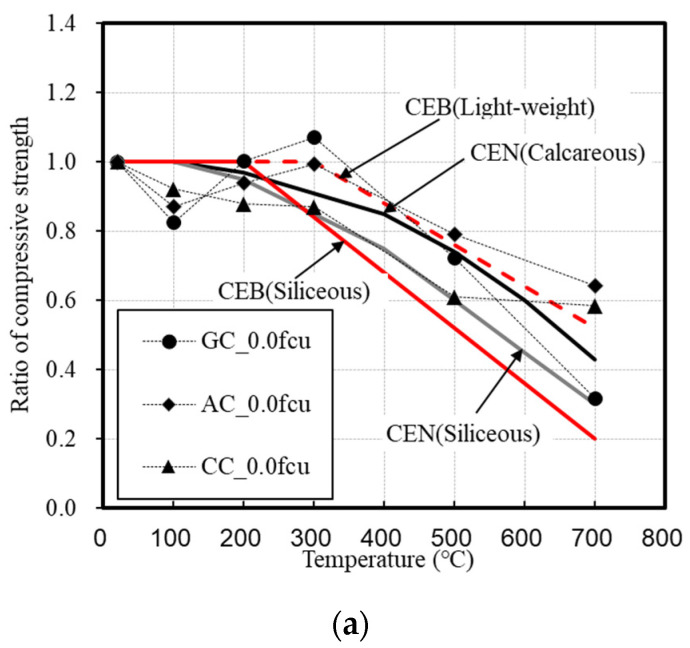

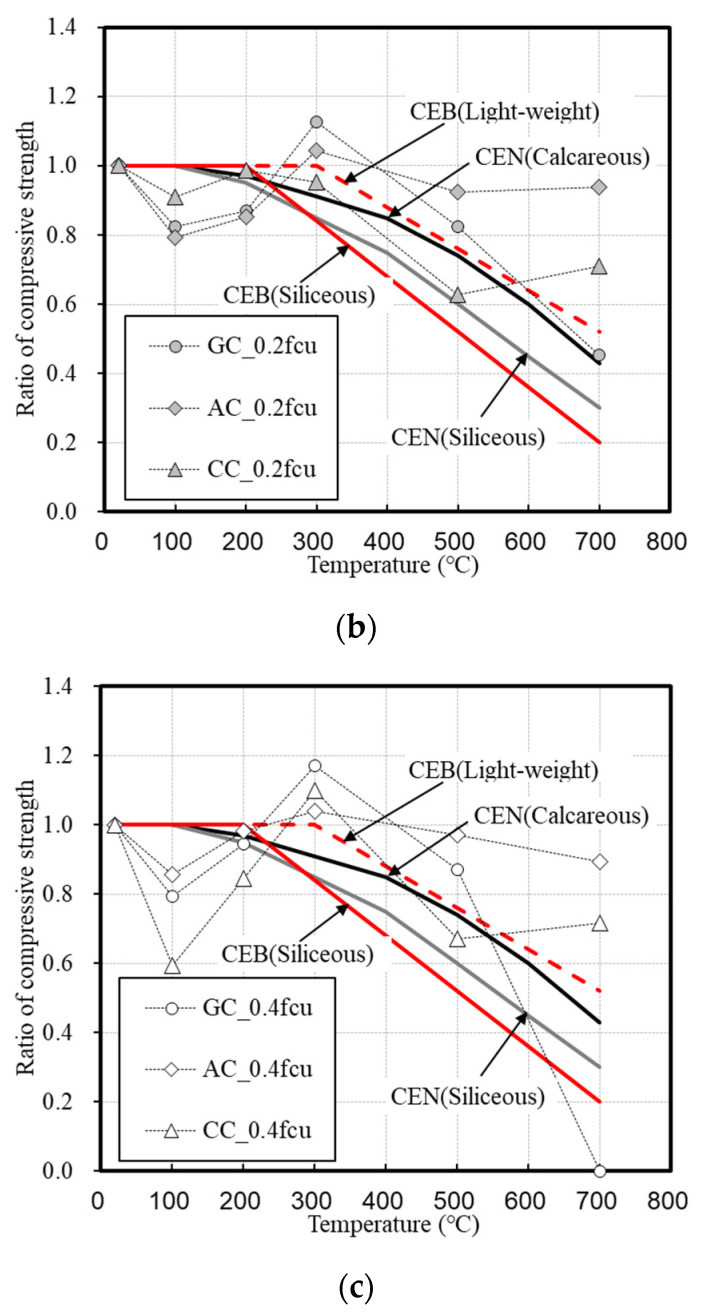
Comparison of experimental data with CEN and CEB codes on compressive strength. (**a**) 0.0 fcu loaded. (**b**) 0.2 fcu loaded. (**c**) 0.4 fcu loaded.

Figure 8 shows the comparison of experimental data with the CEN and CEB codes regarding compressive strength. Under the loading condition of 0.0 fcu, the residual compressive strength of the concrete that used the natural and lightweight aggregates was similar to that of the CEN and CEB codes [11,12]. Under those of 0.2 and 0.4 fcu, the compressive strength was clearly higher than those given in the CEN and CEB codes for all specimens. In the case of the concrete that used lightweight aggregates, the AC exhibited a larger difference. Even though the loading condition increased from 0.2 to 0.4 fcu, there was no significant difference in the residual compressive strength. In the case of the GC, specimen fracture was observed during heating.

A higher value was obtained in this study compared with the CEN and CEB codes because the deformation was controlled according to the load. Because most of the data used in the CEN and CEB codes are experimental results obtained under unstressed conditions, deformation control according to the load increases the residual rate of compressive strength.

### 3.3. Elastic Modulus with Loading and Heating

**Figure 9:**
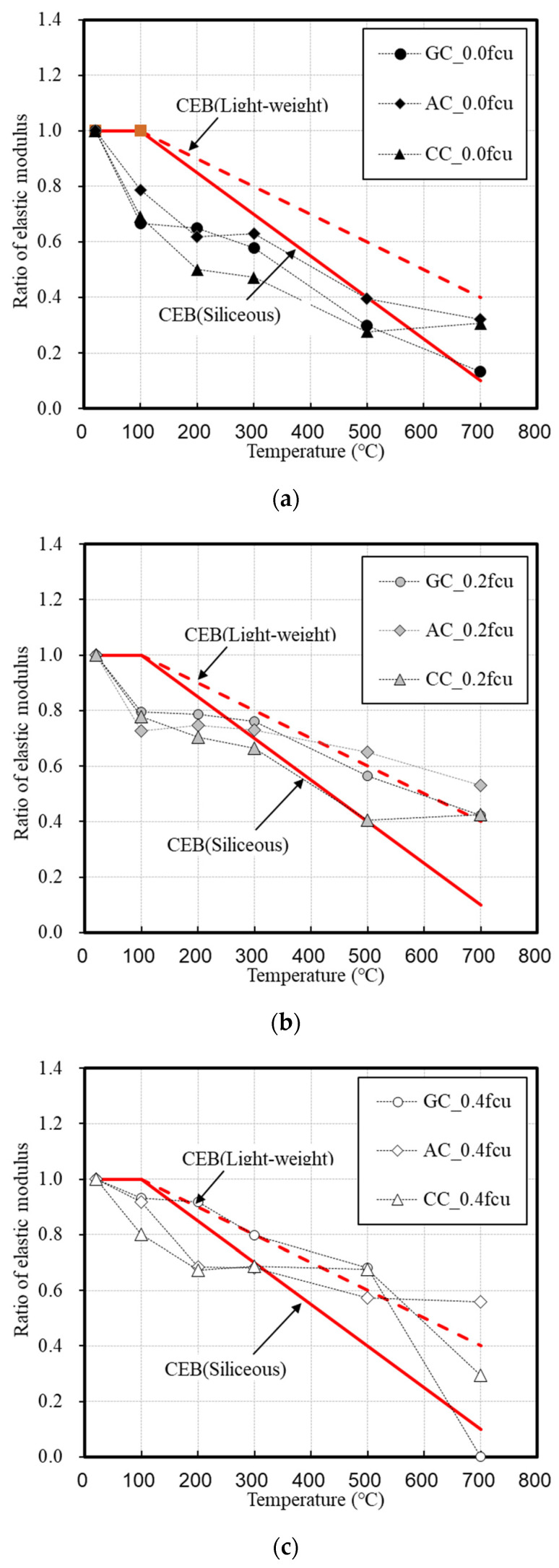
Comparison of experimental data with CEN and CEB codes on elastic modulus. (**a**) 0.0 fcu loaded. (**b**) 0.2 fcu loaded. (**c**) 0.4 fcu loaded.

Figure 9 shows the comparison of the experimental data with the CEN and CEB codes regarding the elastic modulus.

Under the loading condition of 0.0 fcu, the residual elastic modulus of the concrete containing natural and lightweight aggregates tended to be slightly lower than those of the CEN and CEB codes. In particular, the concrete containing lightweight aggregates exhibited a large difference of 40–50%.

When loads of 0.2 and 0.4 fcu were applied, the elastic modulus tended to decrease regardless of the aggregate type, and a tendency similar to that of the CEN and CEB codes was observed.

For the AC, the reduction of the elastic modulus was relatively high, unlike the high residual compressive strength of concrete, but a high value of approximately 60% of the strength at room temperature was observed at 700 °C. The CC showed an increasing difference as the temperature increased from 100–500 °C in a similar manner to the compressive strength tendency, and the residual elastic modulus was also as low as 20% at 700 °C.

Overall, the elastic modulus rapidly decreased in the temperature range of 500–700 °C under the unloaded condition. Under loaded conditions, however, it was approximately 20% higher than that under the unloaded condition. This indicates that thermal strain can be effectively controlled by the binding force in maintaining elastic deformation.

### 3.4. Strain with Loading and Heating

**Figure 10:**
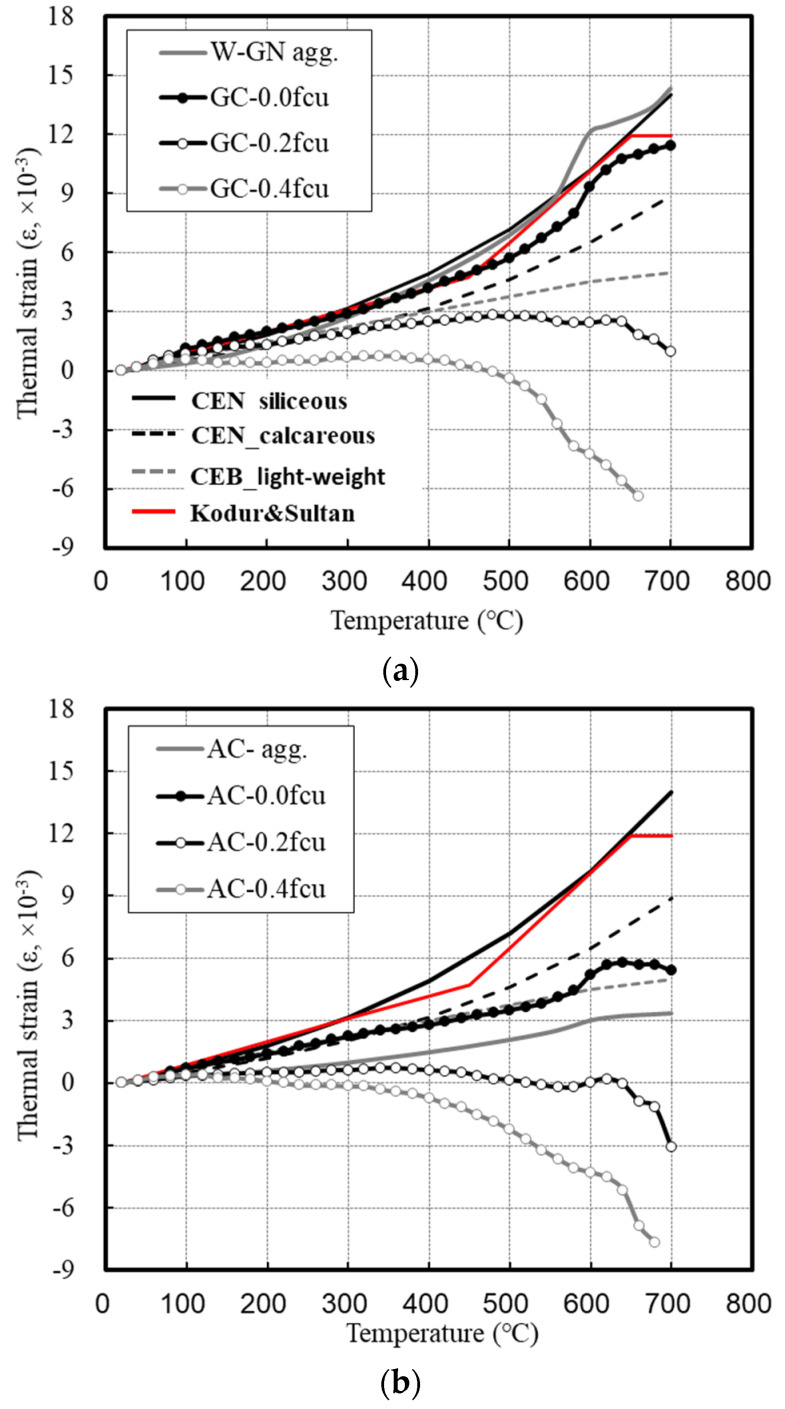

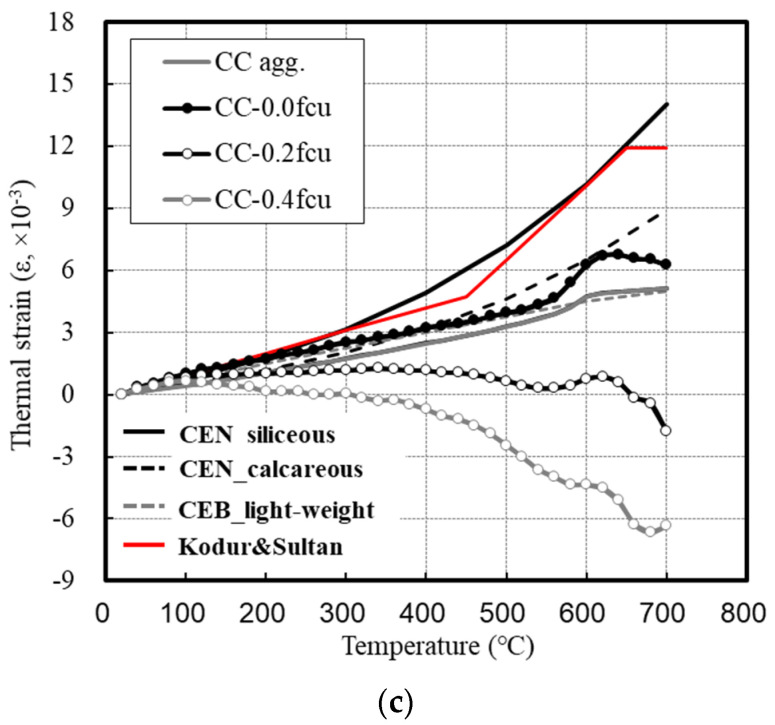
Strain of concrete with heating and loading conditions. (**a**) GC. (**b**) AC. (**c**) CC.

Figure 10 shows the strain of concrete under heating and loading conditions.

Under the loading condition of 0.0 fcu, the GC continuously showed an increased deformation as the temperature increased, and its strain reached 11 (ε, ×10−3) at 700 °C. A comparison with the CEN and CEB codes shows that the actual measurements were slightly different between 600 and 700 °C. In addition, the thermal strains of concrete presented by the CEN are those of the concrete that used silica and calcium aggregates [11,12], and values of the GC lie between them. This is similar to the results of Kodur and Sultan [21].

The main components of the granite aggregate used in this study were quartz and plagioclase. It is estimated that slight differences occurred because silica (SiO_2_), the main component of quartz, has a higher composition ratio than that of calcium (Ca).

The thermal strain of the lightweight aggregates was specified in the CEB code as expanded clay. Under the loading condition of 0.0 fcu, the deformations of the AC and CC tended to be significantly lower than in the CEB code values. The lightweight aggregates used in this study were ash + clay and clay, whose main component was silica. They have many pores inside owing to the high temperatures and expansion during their production. In addition, it is estimated that differences occurred because the materials that constitute the aggregates have different thermal properties.

At 700 °C, the thermal strain of the AC was 6 (ε, ×10^−3^), which was 50% of that of the GC and more than twice as high as that of the aggregate (3 (ε, ×10^−3^)). This appears to be because 7% SF was substituted to develop a certain strength, instead of cement.

Under the loading conditions of 0.2 and 0.4 fcu, the thermal strains of all the concrete specimens were significantly under control. For the GC, the strain continuously increased until reaching 300 °C and converged to 3 (ε, ×10^−3^) after reaching 300 °C under a load of 0.2 fcu. Under the loading condition of 0.4 fcu, the strain slightly increased or remained constant until 400 °C but sharply dropped above 500 °C, which led to failure at 700 °C.

Unlike the GC, the AC and CC maintained their specimen geometry before heating without any thermal strain under the loading condition of 0.2 fcu. However, under a load of 0.4 fcu, they rapidly contracted and exhibited increased deformation after reaching 300 °C. Above 700 °C, the GC did not fail.

When unloaded, concrete shows the formation of macro-cracks between the aggregate and paste at 100 °C or higher [16,30,31]. In addition, the primary decomposition of Ca (OH)_2_ occurs at 538 °C or higher, and secondary decomposition occurs up to 710 °C, thereby causing matrix collapse [32].

In contrast, the loading condition can control the thermal expansion of concrete and delay the matrix collapse. Hertz [16] reported that the thermal expansion of aggregate and change in its condition vary depending on the applied load, and the strength is increased by approximately 25% compared to that in an unloaded condition owing to the control of thermal strain when 25–30% of the preloading condition is applied.

However, above 500 °C, the thermal expansion of granite significantly increases, thereby increasing the thermal stress at the aggregate-paste interface. This accelerates the collapse of the concrete matrix owing to the increase in internal cracking, and a rapid increase in shrinkage leads to the failure of the GC.

It was confirmed that AC and CC have small initial expansion due to the small expansion stress of the aggregates, and they are significantly affected by shrinkage due to their low aggregate strength. The concrete containing lightweight aggregates did not fail due to shrinkage under load because the aggregate and paste exhibited integrated behavior owing to the small thermal stress at their interface.

**Figure 11:**
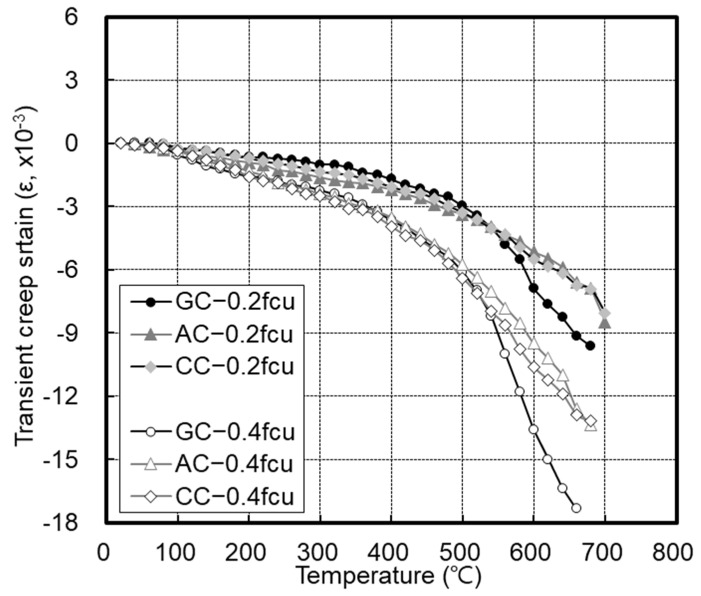
Transient creep strain.

Figure 11 shows the transient creep strain, which continuously increased with temperature, and rapid shrinkage was observed at 500 °C. Therefore, the transient creep showed similar patterns below 500 °C because the residual strength was secured, but it sharply increased above 500 °C owing to the reduced residual strength.

### 3.5. Influence of Aggregates and Loading on the Mechanical Properties of Concrete

The results of this study indicate that the strength of concrete can be secured by controlling its deformation under loaded conditions.

**Figure 12:**
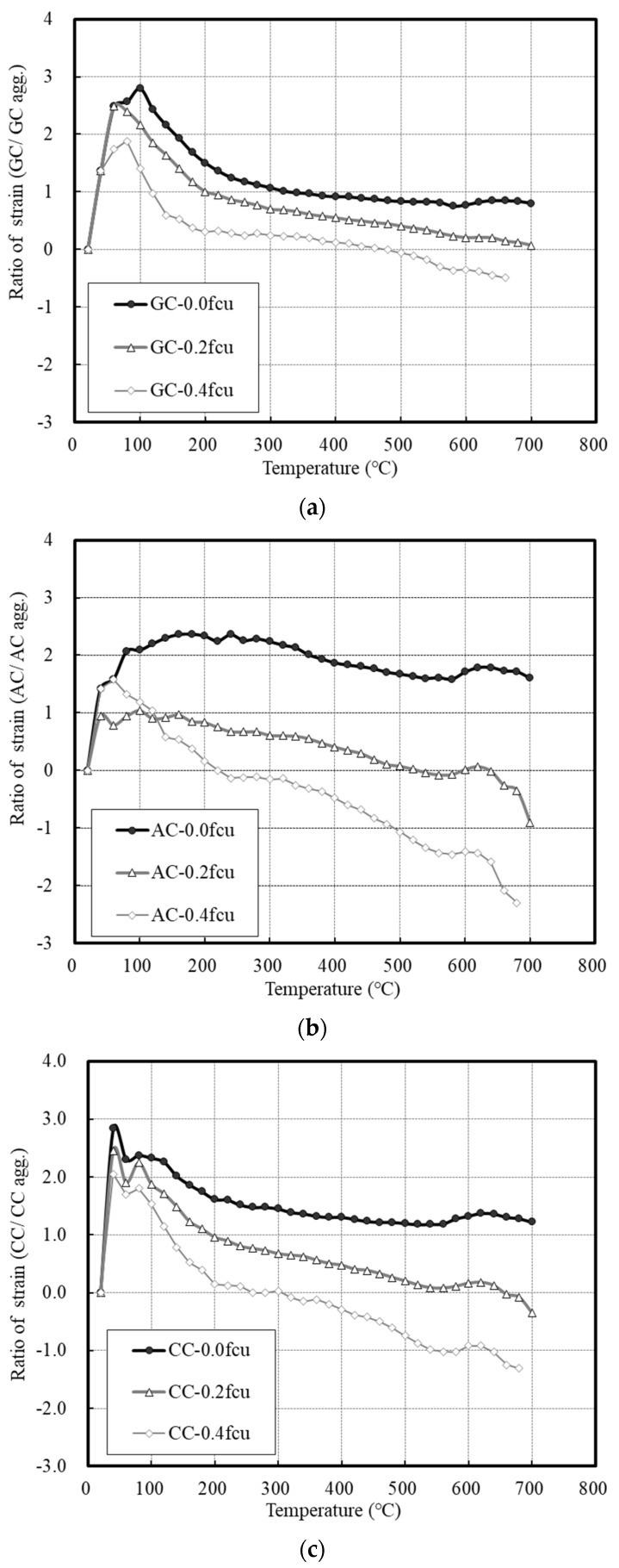
Ratio of strain with heating and loading conditions. (**a**) GC. (**b**) AC. (**c**) CC.

Figure 12 shows the ratio of the strain under heating and loading conditions. Under the loading condition of 0.0 fcu, the aggregate expanded one to three times its original length in the 100–300 °C temperature range, and similar patterns of expansion of the aggregate were observed after reaching 300 °C. The expansion increased in the 100–300 °C range owing to the combined effects of the vapor pressure and thermal stress.

As the applied load increased, the strain ratio decreased. However, the GC expanded owing to the high thermal expansion of the aggregate itself despite the shrinkage and offset effect caused by the applied load. When a load of 0.4 fcu was applied, rapid shrinkage occurred at temperatures higher than 540 °C, which was found to be large despite the continuous thermal strain of the aggregate.

For the AC, the ratio of strain continuously converged to two even under the condition of 0.0 fcu. The AC exhibited a high shrinkage tendency under loading conditions of 0.2 and 0.4 fcu. This is because the strength of the aggregate was relatively low.

The CC exhibited a behavior that was slightly higher than the thermal expansion of the aggregate even under the condition of 0.0 fcu. As the loads of 0.2 and 0.4 fcu were applied, the ratio of strain exhibited a strong shrinkage, and an approximately 1.5 to 2.5 times higher shrinkage tendency compared to the thermal strain of the aggregate was observed at 700 °C.

Deformation at high temperatures can be inhibited under loading conditions of 0.2 to 0.4 fcu, and it was confirmed that the residual strength and deformation control effect were larger as the thermal strain of the aggregate decreased. Therefore, it can be said that the residual strength of concrete increases if the thermal strain of the aggregate is small, and the thermal expansion stress can be controlled according to the load.

The CC containing lightweight aggregate, however, exhibited a tendency similar to that of the GC. Thus, it may have somewhat uncertain aspects that cannot be explained by the thermal strain control effect of loading. Therefore, further analysis is required based on various factors, such as cracks at the interface between the aggregate and paste, deformation under the maximum load, and shrinkage under load.

## 4. Conclusions

The main conclusions of this study are as follows:

1. The loaded conditions were confirmed to be effective in maintaining the elastic behavior of concrete specimens by inhibiting the reduction in the stress–strain slope, and lightweight aggregates with a small coefficient of thermal expansion are more affected by such conditions.
2. The residual compressive strength of the concrete tended to increase as the thermal strain of the aggregate decreased. In the case of lightweight aggregates, the residual compressive strength may vary depending on the chemical composition, despite the small thermal strain. In addition, the residual compressive strength under loaded conditions was higher compared to that at 0.0 fcu, but there was no significant difference at 0.2 fcu or higher.
3. The elastic modulus of the concrete showed a large reduction, unlike the residual compressive strength, but it was approximately 20% higher under loaded conditions. Therefore, the control of deformation by loading decreases the reduction rate of the elastic modulus.
4. Under the loading conditions of 0.2 and 0.4 fcu, the thermal strain of all concrete specimens was significantly under control. Under loaded conditions, however, deformation is significantly inhibited because lightweight aggregates have a strong tendency to shrink, which may have a significant influence on the residual strength of concrete.

In the scope of this study, the effect of the thermal properties of aggregates on the mechanical properties of concrete was investigated under loading and heating conditions. The experimental results confirmed that the concrete containing lightweight aggregates with a relatively small thermal strain had a higher residual compressive strength and residual elastic modulus than that containing natural aggregate. However, lightweight aggregates may exhibit behaviors similar to those of natural aggregates, depending on their chemical composition. Therefore, further analysis of the state of the interface between the aggregate and paste and behavior of the aggregate in future studies is necessary.

## Figures and Tables

**Table 1:** Chemical properties of cement, silica fume and coarse aggregate.

Chemical Composition (wt.%)	OPC	SF	Coarse Aggregate		
			Granite	Ash-Clay	Clay
SiO_2_	21.65	90.00	72.0	72.0	69.20
Al_2_O_2_	5.41	1.50	15.50	13.60	16.70
Fe_2_O_3_	3.24	3.00	1.80	3.10	7.50
CaO	63.37	2.00	2.30	1.50	0.70
MgO	2.28	0.30	0.10	0.70	1.40
Na_2_O	-	-	4.42	1.50	0.90
K2O	1.04	-	3.10	1.20	1.80
SO_3_	2.04	0.40	-	-	-
Loss of ignition	0.97	3.00	-	-	-

**Table 2:** Physical properties of material.

Physical Properties	OPC (1)	SF (2)	Coarse Aggregate		
			Granite	Ash-Clay	Clay
Specific surface (cm^2^/g)	3160	200,000	-	-	-
Density (kg/m^3^)	150	2200	2670	1680	1790
Size (mm)	-	-	20	13	13
Absorption (%)	-	-	1.0	15.2	17.4

(1) OPC: ordinary Portland cement; (2) SF: silica fume.

**Table 3:** Experimental plan.

Specimen ID.	Water-Binder	Coarse Aggregate Type	Loading Condition	Heating Method	Test Item
GC (1)	0.35	Granite	0.0 fcu 0.2 fcu 0.4 fcu (4)	100 ~ 700 °C (0.77 °C/min, 1 °C/min.)	▪Stress–strain ▪Compressive strength (MPa) ▪Elastic modulus (GPa) ▪Thermal strain ▪Transient creep
AC (2)	0.33	Ash-clay			
CC (3)	0.33	Clay			

(1) GC: granite concrete; (2) AC: ash-clay concrete; (3) CC: clay concrete (4) 0.4 fcu: load condition of 0.4× compressive strength at room temperature (20 ± 2 °C).

**Table 4:** Proportion of the concrete mixtures and properties of the fresh concrete.

Concrete Type	GC	AC	CC
Water/cement	0.35	0.33	0.33
Water (kg/m^3^)	165	155	155
Cement content Type 1 (kg/m^3^)	470	432	432
Silica fume (kg/m^3^)	-	38	38
Fine aggregate (kg/m^3^)	692	687	687
Granite (kg/m^3^)	1075	-	-
Ash-clay (kg/m^3^)	-	676	-
Clay (kg/m^3^)	-	-	720
Unit weight(kg/m^3^)	2410	1958	2031
Slump (mm)	190	180	175
Air content (%)	3.3	3.5	3.6
Hardened concrete			
Compressive strength (MPa)			
28 days	55.8	63.6	50.0
180 days	60.2	65.3	52.0

**Table 5:** Test methods for estimating fresh and hardened properties of concrete.

Type	Evaluation Parameter	Test Method
Fresh and mechanical property analysis	Slump (mm)	ASTM C143 [22]
	Air content (%)	ASTM C231 [23]
	Compressive strength (MPa)	ASTM C873 [24]
		ASTM C39 [25]
	Elastic modulus (GPa)	ASTM C469 [26]
	Thermal strain, transient creep strain	RILEM TC 129-MHT [27,28,29]

## Data Availability

The data presented in this study are available on request from the corresponding author.
